# The Evolving Role of Area Agencies on Aging Through the COVID-19 Pandemic and Beyond

**DOI:** 10.1093/geroni/igab046.1794

**Published:** 2021-12-17

**Authors:** Traci Wilson, Elizabeth Blair

**Affiliations:** 1 USAging, Washington, District of Columbia, United States; 2 n; 4 a, Washington, District of Columbia, United States

## Abstract

Area Agencies on Aging (AAAs) have played an important and well-documented role in meeting the nutritional and wellness needs of older adults during COVID-19. To better understand the continued impact of COVID-19 pandemic on AAA services, partnerships, and clients, the National Association of Area Agencies on Aging surveyed the nation’s 618 AAAs in February 2021, with a 27% response rate. As a result of COVID-19, most AAAs reported both increased numbers of new clients and needs of existing clients; three-quarters of AAAs developed new external partnerships; and over half are implementing strategies to address equity and inclusion regarding their services and clients. Nearly 80% of AAAs are involved with COVID-19 vaccine outreach and delivery, from scheduling appointments to administering the vaccine to homebound clients. We will describe these and other transformations of services, partnerships, and client needs; discuss challenges and opportunities; and provide examples and video vignettes from AAA directors.

